# Employee Mental Health During COVID-19 Adaptation: Observations of Occupational Safety and Health/Human Resource Professionals in Ireland

**DOI:** 10.3389/ijph.2022.1604720

**Published:** 2022-08-09

**Authors:** Yanbing Chen, Carolyn Ingram, Vicky Downey, Mark Roe, Anne Drummond, Penpatra Sripaiboonkij, Claire Buckley, Elizabeth Alvarez, Carla Perrotta, Conor Buggy

**Affiliations:** ^1^ School of Public Health, Physiotherapy and Sports Science, University College Dublin, Dublin, Ireland; ^2^ Health Service Executive, Dublin, Ireland; ^3^ School of Public Health, University College Cork, Cork, Ireland; ^4^ Department of Health Research Methods, Evidence and Impact, McMaster University, Hamilton, ON, Canada

**Keywords:** mental health, COVID-19, fatigue, occupational health, employee wellbeing, work adaptation

## Abstract

**Objectives:** This study aims to understand mental health issues among Irish employees arising from COVID-19 adaptation from the perspective of Occupational Safety and Health (OSH) and/or Human Resource (HR) professionals.

**Methods:** Fifteen focus groups including 60 OSH/HR professionals from various sectors were conducted covering four predetermined themes. The data were transcribed verbatim, with transcripts entered into Nvivo for thematic analysis incorporating intercoder reliability testing.

**Results:** The mental health impacts among employees are identified from three stages: pre-adaptation, during adaptation, and post-adaptation. Most issues were reported during the second stage when working conditions dramatically changed to follow emerging COVID-19 policies. The identified mental health support from participating organizations included providing timely and reliable information, Employee Assistance Programme (EAP), informal communication channels, hybrid work schedules and reinforcement of control measures.

**Conclusion:** This study explores the challenges facing employees during the different stages of COVID-19 adaptation and the associated mental health impacts. Gender’s influence on mental health consultations should be considered when planning for public health emergencies, and further research conducted in male dominated industries.

## Introduction

The emergence of COVID-19 has resulted in workplace adaptations or operational/environmental changes at workplace worldwide. To mitigate the transmission risk, employees had to rapidly adapt to relevant public health measures at various points during the pandemic including, working from home (WFH) or in the case of essential workers to social distancing, the use of Personal Protective Equipment (PPE), or frequent testing often without adequate psychological adjustments. Adverse psychological outcomes were observed from the beginning of the pandemic when employees, in addition to adjusting to public health measures, were challenged by mandatory quarantine, school closures, unexpected unemployment, and related uncertainties [1, 2]. Factors contributing to adverse mental health impacts on employees during COVID-19 were summarized by a review study [3] from several aspects. Specifically, heightened perception of COVID-19 contagion risk has been identified as a predictor of poor mental health. The perceived risk of being infected at work which varies depending on demographics [4] is positively associated with emotional exhaustion [5]. Also, individuals may fear for their family members’ health and safety [6]. Infodemic versus the unknown [7] contributes to pandemic fatigue as individuals are constantly exposed to an overload of rapidly changing COVID-19 information [8]. Quarantine and confinement slow the spread of infectious disease while potentially increasing risk of anxiety and depressive symptoms [1]. Lack of social interactions between colleagues can increase employee stress levels [1]. Stigma and social exclusion are frequently observed among healthcare workers leading to psychological distress and depression [9]. Additionally, individuals experiencing loss of income are reportedly more distressed and in poorer health [6].

Despite the variety of published research, there is an ongoing need for further research in this area that focuses on employees from an occupational adaptation perspective [10], since mental health impacts of COVID-19 may take weeks or months to become fully apparent with possible long-term occupational health ramifications well into the future [11]. This paper aims to explore how workplace COVID-19 adaptations contribute to employee mental health pressures and how organizations can alleviate such issues while maintaining a safe workplace environment. In general, employee welfare is in the realm of Resource (HR) management in industries in Ireland, while Occupational Safety and Health (OSH) management focuses on specific measures implementation in relation to employees’ safety, health and wellbeing. Compared to HR professionals who usually liaise with upper management and oversee allocation of resources, the role of OSH may be somewhat neglected especially in low-risk work settings. However, since some organizations were lack of OSH professionals during the COVID-19 pandemic, Human Resource (HR) professionals consequently played a similar role to be responsible for employee health and wellbeing, especially in small and medium sized enterprises. Thus, OSH/HR professional group possesses key insight into employee adaptation during COVID-19 and occupational strategies for mental wellbeing. By conducting focus groups with this cohort, this study contributes to understanding the mental health impacts arising from the workforce adaptation to the new safety measures implemented due to COVID-19.

## Methods

### Recruitment of Participants

To gain in-depth insight from the perspectives of OSH/HR professionals, focus group was the chosen data gathering method. In line with COVID-19 regulations, the fifteen focus groups were conducted via online ZOOM^TM^ meetings between April and May 2021. The participants were mainly recruited through the Centre for Health and Safety alumni network. Participants from similar sectors were designated to the same group so that peers could exchange shared experiences. For example, Focus Group (FG)3 participants were from Biopharmachem sectors; FG4 participants worked in Transportation/Logistics.

### Data Collection and Instruments

The data collection stopped when 60 participants were interviewed and data saturation was reached [12]. All ZOOM^TM^ meetings had four sets of five-question polls built in around specific themes and the participants took part in them to supplement the qualitative data. To ensure communication efficiency, the number of participants in each group was limited to four to six per session [13]. Focus group interview protocol related to four predefined topics (Table 1): preparedness and support; actions and impact on the organization; impact on workers; and lessons to be kept moving forward. The protocol was reviewed by multi-disciplinary experts from the research team (e.g., backgrounds in OSH, medicine, psychology and public health). A 2-hour pilot test was conducted within the research team on ZOOM^TM^ prior to data collection to refine the protocol. All focus groups were conducted by CB to ensure consistency, with YC and MR in attendance to observe and note take as a backup in case the recording became corrupt or unusable, which also complemented the ZOOM^TM^ recordings (as ZOOM^TM^ can only record the speaking person even if there are more than one participant in the meeting.

**TABLE 1:** Focus group interview questions, workplace adaptation to COVID-19, Ireland, 2021.

Theme	Questions
1. Preparedness and support	Prompt Questions
	Do you feel your Organization was prepared for COVID19 and understood the risks associated with it prior to the commencement of lockdown?
	Do you feel you personally professionally were prepared for COVID19 before it emerged in Ireland?
	Do you now consider OSH professionals to be part of public health also?
	Prior to COVID-19 was there an active/visible occupational health function in or for your Organization?
	Prior to COVID-19 did you or your Organization ever have to interact with the HSE or public health division in the Department of Health?
	When lockdown began can you describe the processes you were involved in to protect your workers?
	Potential Follow Up Questions
	What was your primary source of information on how to manage the crisis?
	Do you feel management understood the importance of your OSH work in responding to the pandemic?
2. Actions and impact on the Organization	Prompt Questions
	What was the immediate impact on your workplace when lockdown began?
	How did your Organization adapt with or to changing working conditions?
	How frequently did you have to update your adaptation plans?
	At any time did you have to interact with the national contact tracing team?
	Did you have enough resources to do your job effectively?
	Potential Follow Up Questions
	What OSH professional resources did you access to help you develop adaptation plans?
	What resources could you have used to help you more?
3. The impact on workers	Prompt Questions
	Did you have workers fall ill from COVID-19?
	From your observations how do you think your colleagues have responded to the adaptations made to keep them safe from COVID19?
	Adapting requires behaviour change, have you been able to observe such behavioural changes in your colleagues over the last year?
	As the year has progressed do you see any fatigue in your colleagues directly from the changed working conditions?
	What do you think is leading to that fatigue in your colleagues?
	What other aspects of the pandemic do you think are influencing your colleagues’ behaviour?
	Have any colleagues indicated mental health issues arising from our current situation?
	Potential Follow Up Questions
	Is the behavioural change observed positively or negatively by your colleagues?
	What do you think could be done to alleviate fatigue from workplace adaptation?
	Did any of the workers in your Organization lose family members to COVID-19?
4. The positives and negatives of the last year and what would you keep moving forward	Prompt Questions
	How do you think COVID19 has impacted your colleagues’ knowledge and perceptions of OSH?
	What do you think was the most impactful action you were able to take for your colleagues this last year?
	What do you think the outcome of the pandemic will be for how OSH is managed in your Organization moving forward?
	Is there anything more you think you could have done in the last year?
	What would you do differently in hindsight?
	What (if any) will you bring forward into your OSH management when the pandemic is over?
	Potential Follow Up Questions
	What personally helped you rise to the challenges of the last year?
	What do you think was the biggest challenge to deal with?
	Do you have any worries for the long-term impacts on your colleagues?
Exit question	Is there anything else you would like to say about your experience on dealing with COVID-19 in your workplace?

### Data Analysis

The transcript of the audio recording were generated by ZOOM^TM^, which were verbatim corrected by the researchers following a playback of the original audio files downloaded. Each of the focus group transcripts contained approximately 20,000 words. To align with ethical requirements, participants were assigned pseudonyms according to their working sector after de-identification. To achieve the purpose of this study of understanding the mental health impacts caused by COVID-19 adaptation, two themes were predetermined after the researchers familiarizing themselves with the data: 1) key mental health impacts reported and 2) the related supports from the organization. Following a conventional content analysis approach [14], open coding of data was then conducted under each of themes by highlighting the exact words used by the participants to capture key thoughts or concepts. Reflexive notes were also made as the inductive coding progressed, and the codes were subsequently categorized into meaning groups based on the links between them. For example, based on the characteristics of emotions emerged during different phases of COVID-19 pandemic, the mental health related issues identified were sorted into three groups: pre-adaptation, during adaptation and post-adaptation. The codes/themes were consistently refined through critical dialogues between the researchers, and the analysis was subsequently adjusted deductively after the agreement reached on each update. Through such an iterative process, the final themes/subthemes were thus conceptualized as presented in the result section [15].

### Rigor and Trustworthiness

**FIGURE 1:**
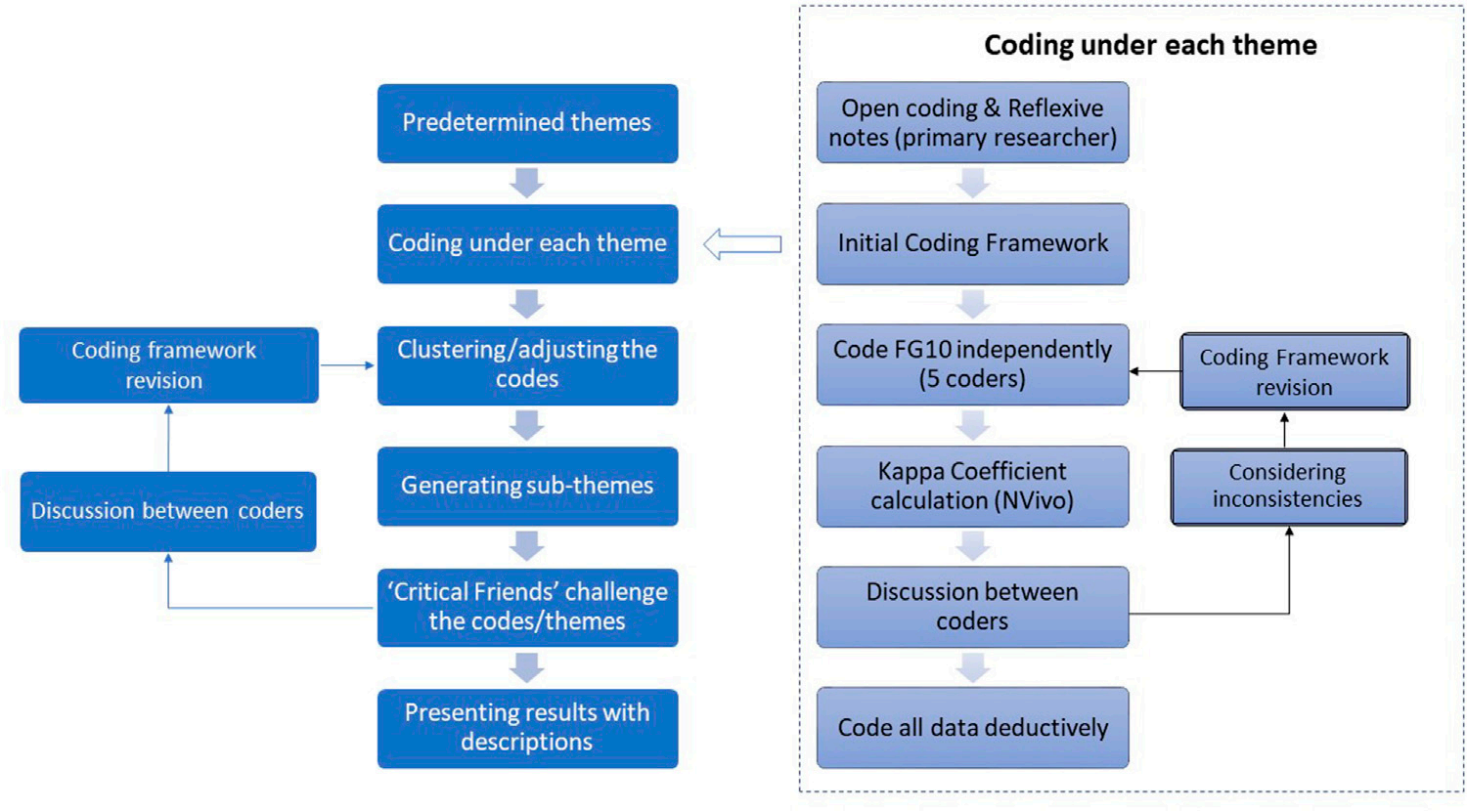
Data analysis flowchart relevant to employee mental health, workplace adaptation to COVID-19, Ireland, 2021.

Rigor and trustworthiness have been considered throughout the study [16]. First, credibility was ensured by data collection triangulation (focus group interviews and quantitative poll questions) and researcher triangulation when analyzing the data. For example, the coding of all qualitative data was completed by five independent coders after an intercoder reliability (ICR) assessment [17]. Specifically, the initial coding round was deductively completed by the primary coder YC, who color coded transcripts in Microsoft Word based on the four topics pre-identified in the protocol. During this process, YC took reflective notes, proposed sub-themes within each category, and chose a transcript (FG10) for ICR assessment. While coding the FG10 transcript, YC developed a primary coding frame by inductively creating an extensive set of descriptive codes under each sub-theme using Nvivo. To assess the level of agreement, coders coded the same part of the transcript independently using the primary coding frame. ICR was calculated based on Cohen’s kappa coefficient using the “coding comparison” function in NVivo. After discussing divergence and refining the themes/codes, remaining transcripts were assigned to coders for thematic analysis. Relevant findings from other research are also discussed as a triangulation. Second, thick descriptions of the theme identified were provided which enable the readers to evaluate the possibility of transferability of the study findings. Finally, the dependability was achieved as the research procedures were transparent (Figure 1) and clearly documented. Therefore, the confirmability was deemed established as the interpretations were cross-checked by the researchers who have multidisciplinary backgrounds in the team.

## Results

**FIGURE 2:**
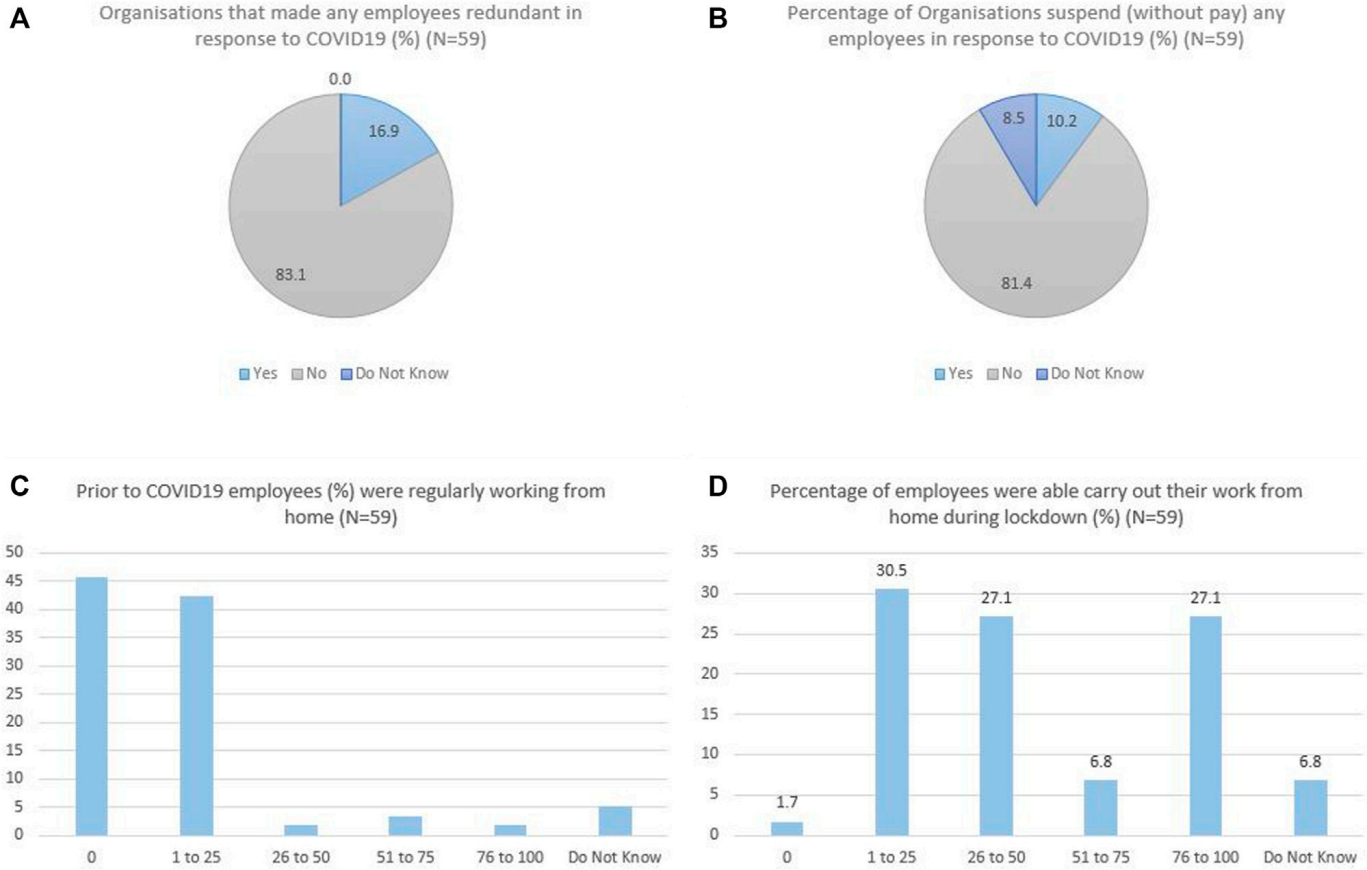
Poll results relevant to employee mental health, workplace adaptation to COVID-19, Ireland, 2021.

The participants’ information including overall gender distribution, type of industries represented and size of their organizations etc. are presented in Table 2. This section focuses on the mental health aspects of the findings, which frequently were discussed by the participants during the study. The results are displayed around mental health impacts on workers, the impacts on adaptive behaviors, and mitigation strategies, with simplified direct quotes from the participants. More detailed narratives are exemplified in Tables 3, 4 to provide richer descriptions and relevant poll results were also provided in Figure 2 to facilitate understanding of the narratives.

**TABLE 2:** Participants’ information, workplace adaptation to COVID-19, Ireland, 2021.

Participant pseudonyms	Organization sector	Industry	Organization size
Biopharmachem 1	Manufacturing	Pharmaceutical/Bioscience	Large
Biopharmachem 2	Manufacturing	Pharmaceutical/Bioscience	Large
Biopharmachem 3	Manufacturing	Pharmaceutical/Bioscience	Large
Biopharmachem 4	Manufacturing	Chemicals/Pharmaceutical	Large
Construction 1	Construction	Building/Telecommunications	Large
Construction 10	Construction	Construction	Large
Construction 11	Construction	Construction	Medium
Construction 2	Construction	Engineering Consultancy	Large
Construction 3	Construction	Engineering Consultancy	Medium
Construction 4	Construction	Construction	Large
Construction 5	Construction	Construction	Small
Construction 6	Construction	Construction	Large
Construction 7	Construction	Construction	Large
Construction 8	Construction	Construction	Large
Construction 9	Construction	Construction	Medium
Consultant 1	Administrative Support	OSH Management Consultancy	Small
Consultant 2	Administrative Support	OSH Management Consultancy	Small
Consultant 3	Administrative Support	OSH Management Consultancy	Small
Consultant 4	Administrative Support	OSH Management Consultancy	Small
Consultant 5	Administrative Support	OSH Management Consultancy	Small
Consultant 6	Administrative Support	OSH Management Consultancy	Medium
Financial 1	Financial	Banking	Large
Financial 2	Financial	Insurance	Medium
Financial 3	Financial	Health Insurance Provider	Medium
Healthcare 1	Public Sector	Major Hospital	Large
Healthcare 2	Public Sector	Educational/Care	Medium
Healthcare 3	Public Sector	Healthcare Provision	Large
Healthcare 5	Public Sector	Major Hospital	Large
Healthcare 7	Accommodation/Hospitality	Private Nursing Home Management	Large
Healthcare 8	Accommodation/Hospitality	Private Nursing Home Management	Large
Hospitality 1	Accommodation/Hospitality	Hotel	Medium
Infrastructure 1	Electricity/Gas	Telecommunications Support	Large
Infrastructure 2	Electricity/Gas	Electricity	Large
Infrastructure 3	Electricity/Gas	Energy Infrastructure	Large
Infrastructure 4	Public Sector	Infrastructure Division	Large
Infrastructure 5	Public Sector	Public Passenger Management	Large
Infrastructure 6	Public Sector	Production Planning Division	Large
Infrastructure 7	Transportation/Logistics	Airport Management	Medium
Infrastructure 8	Electricity/Gas	Electricity	Large
Infrastructure 9	Electricity/Gas	Oil Refinery	Medium
Infrastructure 10	Public Sector	Transport Advisory Agency	Medium
Local Authority 1	Public Sector	Local Authority	Large
Local Authority 2	Public Sector	Local Authority	Large
Local Authority 3	Public Sector	Local Authority	Large
Local Authority 4	Public Sector	Emergency Service Support	Medium
Local Authority 5	Public Sector	Local Authority	Large
Logistics 1	Transportation/Logistics	Logistics Division for pharmaceutical distribution	Large
Logistics 2	Transportation/Logistics	Waste Management	Large
Manufacturing 1	Manufacturing	Multisector business	Large
Manufacturing 2	Manufacturing	Food manufacturing	Large
Manufacturing 3	Manufacturing	Engineering/construction manufacturing	Small
Manufacturing 4	Mining/Quarrying	Mining	Medium
Manufacturing 5	Manufacturing	Printing	Small
Manufacturing 6	Manufacturing	IT Equipment	Large
National Agency 1	Public Sector	Health Services Oversight	Large
National Agency 2	Public Sector	Government Cross Departmental Office	Medium
National Agency 3	Public Sector	Food Manufacturing Regulation	Large
National Agency 4	Public Sector	Agriculture Client Division	Large
National Agency 5	Public Sector	National Broadcasting/Media	Large
National Agency 6	Public Sector	Emergency Service Support	Large

**TABLE 3:** Identified mental health impacts on employees, workplace adaptation to COVID-19, Ireland, 2021.

Mental health impacts	Description/Sub-theme	Example quote
Pandemic phase: Pre-Adaptation		
Stress	Uncertainty due to unprecedented nature of the pandemic	Construction 5: it was, I found that a very stressful time because you know you’re really, I’ve never experienced a pandemic before. You read about the Spanish flu, but this was something totally different. (FG11)
		Infrastructure 1: They were a little panicked at the start. Were they all going to get it out working on the side of the road or wherever they may have been, and I think the assurances we were given them, and we were explaining how the disease was being transmitted. (FG13)
	Lack of preparedness	Local Authority 1: We were definitely not ready … all queries seemed to come to health and safety, to see what we were doing and what we could do. (FG15)
		Biopharmachem 3: that was never going to be easy and this is just probably a game changer. It’s changed multiple things for the future, so plans are in place to certain extent, and our corporate US would have leveraged plans and then [we] copied them. While you can plan for the scenario, I don’t think it fully, it doesn’t fully psychologically prepare people for something that they don’t ever expect to happen. (FG14)
Panic	Panic behaviors of colleagues	Local Authority 1: It was madness at the start there was, the handling of money, like is that safe, you know there was people bringing in money and putting it in envelopes and locking it in safes and stuff like this totally fishing in the dark at the start. (FG15)
Frustration	Disconnect between expectations from management and realities of the pandemic	Construction 7: I’m just being honest here on that, and what I found frustrating, and I think I remember from the very first announcement, there was [a] Friday afternoon and it meant that you were now in crisis mode on over the weekend and it didn’t allow I don’t think employers anywhere, the opportunity here to plan this during the day, I think the schools lockdown and they got notified in morning, and they were leaving by lunchtime all the parents had to arrive and get them home, but in the workplace, none of that was ever forwarded. (FG14)
		Construction 11: I know definitely before the first lockdown people were really frustrated and upset but basically, none of us knew anything about COVID and what was going to happen, so people were frustrated that they were still working on site when they didn’t feel like they were safe to be working on site. (FG12)
Pandemic phase: During adaptation		
Isolation/loneliness	Working from home and lockdown restrictions lead to social isolation	National Agency 5: it’s mainly from those who are working from home, some of them are, you know they’re single people, they’re living in a bad status or something like that. They haven’t even been there, working from an ironing board if have a laptop. Lockdown has you know, has kept them indoors are restricted to 5K (kilometres). (FG5)
		Consultant 1: For staff, particularly the staff that would be, for example, living on their own anyway, and then COVID came along, so there were socially physically isolated because their social interaction was in the workforce and then that went by the wayside. (FG8)
		Manufacturing 1: we have someone that has a mental health issue, because it is only recently, I mean just really struggling at the moment, but, as well as that there’s a couple of, we have a couple of foreign people that work for us, they’re on their own and they’re going home from work and they’re isolated in their own [accommodation] and really constantly go back to the apartment on their own, where with a roommate they may don’t know or somebody will be shared around. They just stucked [stuck], with no family, something like that, see it’s still we can see it’s tough on them as well. (FG2)
Worry	Uncertainty regarding work projects	Construction 8: I think after the first lockdown, you know when we returned to work there was still a large degree of fear there so there was between operatives working on the ground, you know. I would say, it certainly impacted from the point of view of whether they were unsure about the situation at work, how was it going to affect future projects that they may have had lined up or where projects were put on hold. I think that impacted a lot on people on the ground from you know a lot of people worry about that sort of stuff obviously. (FG12)
	lack of transparent communication from management	Construction 11: since coming back after any of the lockdowns there is a lack of communication on a building site just to say there’s a positive test, there’s a positive case on a building site but it’s not communicated properly and the precautions aren’t communicated properly. People really do get nervous, and they got really scared. Again, especially if they’re living with someone who could be considered a vulnerable person. (FG12)
	Worry of lost income	Manufacturing 2: We also, the nature of our business, we have a lot of people that would [be just] beyond [or] near minimum wage, so they’d be the type that are more inclined to come to work rather than, say, someone that’s on a good wage, because they need the money. (FG2)
	Stress stemming from lack of IT skills	Infrastructure 2: And she happens to live on her own, she’s a cancer survivor and she’s also an asthmatic so you know a lot of a lot of issues that you’d want to be helping people with. And the IT point of it was really stressful for her. (FG4)
Fear	Fear of contracting the virus	Manufacturing 5: They jump out of the way now, when you come towards to me, they kind of ‘stop, here is my space!’ People are more aware now and it’s mad, sometimes even if you’re passing someone on the stairs and they step back to the bottom, to the floor, … you know, like be realistic about themselves, I think that’s probably the thing that people are more aware of ‘whoa, don’t come too close to me.’ (FG4)
	Heighted fear of contracting the virus if vulnerable	Consultant 1: With regards to the seniors, more senior people and my experience in the out, doing training and courses, there’s some of them are fearful, absolutely fearful because they’ve underlying conditions. (FG8)
	Concern of giving the virus to vulnerable loved ones	Local Authority 3: particularly from our side, you’d hear more staff talking about maybe their mother wasn’t well or the father wasn’t well or granny was living in the house, and that they were concerned about that. (FG9)
		Construction 11: Yeah, a lot of people would be far more nervous now about coming into work particularly if they have someone at home that could be considered a vulnerable person, and I also found that their mental health has definitely been affected during COVID I think everyone’s has. (FG12)
Internal conflict	Conflicting physical/social and health-related needs	Consultant 3: I saw my grandmother for the first time in the 3 months at Christmas and I went to give her a hug, and she was actually terrified that I was coming into her personal space like, you know. I thought the hug it was important, but she wanted to get out of my arm straightway, so you know, I’m up [set] at the same time she’s panting for human contact, so it’s having weird effects on people, people want the contact, [but] they are afraid of contact as well. (FG8)
Grief	Overwhelming loss in the healthcare sector	Healthcare 7: It’s like a war zone, it’s it’s devastating. A lot of carers who work they’re not there for the money, they’re genuinely there because they love caring and there are very strong, bonds and relationships between carers and their residents, I mean they’re literally like their grandparents as such. To see staff working homes and they initially are losing 1, 2, 3, 4 not only residents, but sort of their friends, their sort of mentors, their whatever way you’d like to describe relation, it is, it’s just devastating, it is just I mean they are literally just in shock it’s very, very severe. (FG6)
	Lack of ritual or funeral due to COVID-19 restrictions	Manufacturing 4: I know at a conference and I knew a guy here around the meeting. You know, a safety professional, we were still trying to prevent this enemy coming in, coming in the door and I’m shortness are you know, certainly affecting our workforce from, from a health, health perspective, you know I can, enough of them have lost have lost, you know loved ones, through it, as well, which is, which is sad and, and can’t do what the Irish do well as give them a proper, proper burial and funeral etc, etc, but that’s, that’s no, that’s no [funeral]. (FG2)
Pandemic phase: post-adaptation		
Constant fear	Ongoing high alert	Healthcare 5: And I suppose the fear is, if you relax things, even in the workplace too soon, it’s like what they’re saying nationally, then we could have it [COVID-19] back again so if we go back from one chair (employee) to two if some staff aren’t vaccinated is that going to cause a problem, are we going to have transfers again. (FG5)
		National Agency 4: I would say that the whole issue of fear was there, you know that you’re that and that’s tied into doing the right thing… you know that we’re on high alert and it’s ongoing. (FG7)
	Constant barrage of negative media/infodemic	Consultant 6: it’s COVID fatigue that has set in people are sick and tired of the same subject, time after time, and hour after hour, and it’s the same stuff and news, it’s not new information necessarily, it’s just news, so it’s very, very much the same old same old. (FG8)
Fatigue	Fatigue led to inadequate compliance over time with control measures	Manufacturing 6: um again, I think, honestly, I really do think that 80%–90% of people are very, very committed. And there’s just a little bit of fatigue on the facemask wearing as it goes on and goes on people are getting a little bit more tired of it. (FG6)
		Construction 8: I would say there is certainly, you know as time went on a certain level of COVID-19 fatigue set in place, and you know, it’s like anything when people start to get familiar with it and the fear of it gradually, you know died off a little. (FG12)
	Fatigue due to COVID longevity	Infrastructure 6: I think that obviously affects my mental health because I don’t feel good about myself, but I think that’s also in other people, but the fatigue part of it, I think they’re mentally drained of the whole thing. (FG4)
		Healthcare 1: But we’re performing at a much higher base anyway because everyone is dealing with their personal COVID and their changed family circumstances so already they’ve got so many different levels of stress, but not having that time to disconnect and share and offload that piece, absolutely and utterly we’re seeing that [fatigue] (FG5)

**TABLE 4:** Identified solutions to alleviate mental health issues, workplace adaptation to COVID-19, Ireland, 2021.

Solutions for mental wellbeing	Considerations	Example quote
Timely, reliable information from management on COVID-19	• Conflicting information as source of anxiety for employees	Infrastructure 1: They were a little panicked at the start were they all going to get it out working on the side of the road or wherever they may have been, and I think the assurances we were given them, and we were explaining how the disease was being transmitted. (FG13)
	• Employees frustrated by lack of transparent communication on positive cases in the workplace	Infrastructure 7: …and in the air, the fear and the uncertainty of the unknown. And then you know when you see numbers of people that are actually dying from contracting COVID-19, and we would have been busy trying to educate our staff because I suppose, fear sometimes can be a good motivator. So we wanted to educate our staff about the long term impacts of COVID-19 and what the term now is “long COVID.” (FG8)
Employee Assistance Program (EAP)	• Companies usually have EAP to support employees	Construction 6: we had a number of people that had wellbeing, had issues, whether it be financial, emotional and physical from COVID, so we have an EAP within the company and there were a number people who reached out. (FG11)
	• Companies had to upscale EAP resources during the pandemic due to increased demand	Local Authority 1: we have a health and wellbeing unit so we’ve, that was initially set up just before COVID got in it was very much in its infancy, for a large organization, we are way behind the curve on it so we’ve rolled out resilience programs and we’ve rolled out loads of information programs like at the moment we’re running a range of seminars. (FG15)
	• Some programs still in infancy prior to the public health crisis	Manufacturing 4: We certainly, from the mental health side of things, and we, we have seen it’s been quite busy throughout the year and I can’t tell you, for the last 2 months the what the numbers are, it’s very confidential, of course, and stuff is up, we would we would only get a number, as in many, many contactors, we use the EAP program like etc, etc, but we also have a first aid mental health committee here as well. (FG2)
	• Male employees more inclined to seek out female supports	Construction 4: I can give a woman’s perspective which might be different from a man’s perspective. Being a woman in a male dominated industry, I find men come to women’s talks (talk to women). I find my office a little bit like a counselling office at sometimes you know. Man to man, you know less likely. (FG11)
Informal communication channels	• Informal communication can be initiated by OSH professionals	Biopharmachem 1: it was literally you forgot to read the book of how to manage a pandemic through health and safety and no, but I think the key thing was, it was actually [communicate with] more people, as in their emotion and their worries, their fears… Try to support them, so you just you kind of had to sit there and let them just be an ear, more than anything else (FG3)
	• Shift in workplace culture towards emphasis on mental wellbeing as a result of the pandemic	Infrastructure 6: Like I was shouting from the top of a roof [to release my stress], maybe a year ago, or 2 years ago and to be totally honest with you, people call me a soft f* idiot, ‘What are you doing, would you ever just get the job done!’ You know, whereas I can honestly say in, since Christmas four people have contacted me looking for a phone number [for mental health support]. So I think it’s very different in how people now feel about, how people feel they can maybe stand up a little bit more and talk about and how they interact and who they trust to interact with. (FG4)
	• Facilitating opportunities for safe socialization between employees (even virtually) is important	Financial 1: And I said early on it, and I want to stay on this call, and just have a chinwag (chat) and we’ve just talked about the visitor’s procedure, but you know I mean I don’t want to talk about the weather, for a bit and people, oh yeah all right, then yeah, so encouraged doing that and I think that, if anything, that’s what people are missing. It’s great. I mean this sort of thing Skype and zoom and the rest of it. (FG16)
		Local Authority 4: And I do find myself I know these headphones are on me nearly all day, every day, and you do tend to … you know, a conversation that really should only take five minutes ends up taking 25 [minutes] but again back to the informal chats, it’s amazing what can be resolved, you know, rather than an email that’s meant sort of informally, but maybe it's tongue is perceived as being informal and gets people’s back up so. (FG9)
	• Challenging for male employees to open up on mental health but beneficial when they do	Biopharmachem 2: He broke down and cried. And once I got it out of him, he was fine. It was like that it was building up [and] building up until he actually broke down and [he said:] “I just haven’t seen my daughter! I want to see her [but I cannot] until this company gets it right!” And you blame the country for a worldwide pandemic, but you know it was one of those ones that we didn’t consider at all. (FG3)
Hybrid work schedules	• Challenges to working from home emerged over time due to lack of socialization	Construction 8: I think the whole working at home topic is interesting as well, in that I don’t think people you know found that as good as what they thought it would be. A lot of people miss the interaction in instances, I think it has to be, you know, a large majority, people I talk to you know they want the combination, so I think a lot of people will come back to the offices when they have the opportunity to do that as well. (FG12)
	• Conflicting feelings over wanting to return to work for the social aspect and virus-related anxiety	Financial 3: Because it’s not small mental health issues, actually, we found that they have become very unwell you know the people that haven’t been able to cope so it’s just yeah we look forward to, I suppose, being able to give them a little bit more of social activity back in the office but what we’re actually seeing now is that there’s is we look at going back to the offices that there’s an anxiety arising there as well. (FG13)
Reinforcement of control measures	•Reinforcement helps to cope with behavioral fatigue and encourage compliance	Financial 2: And people just I know, for whatever reason, they knew what they needed to do but for one reason or another, at times, there were a bit lax on it had to be reinforced us, you know this is how it’s going to operate and. But, in general I thought, you know yeah very, very few people involved in the first place, but people do get lax over a period of time and they need to be enforced. (FG3)
	•Reinforcement important for reducing employee fears of contracting the virus at work	Manufacturing 1: we have a lot of staff, they didn’t, they didn’t want like we were considered essential straightaway for the, for the maintenance of that sense, but they didn’t want to come into work because they didn’t know what was happening, because they were thinking I’ve underlying conditions, I’ve this and that and I don’t feel safe coming into work yet, so we had to do a lot of convincing to make to lads that the site is safe, we have these controls in place and you’re good to come back now. (FG2)
		Healthcare 3: I made sure I had to protect myself. And I have to supply my PPE if I need it and that’s the way it is. I just had to make sure I was safe. (FG5)

### Mental Health Impacts

The mental health impacts of COVID-19 adaptation on employees are identified from three pandemic stages: pre-adaptation, during adaptation, and post-adaptation. The pre-adaptation phase refers to the period between December 2019 (first COVID-19 case was officially reported in China) to late March 2020 (public health measures to delay the spread of COVID-19 announced in Ireland). “During adaptation” is considered from April 2020 towards the end of the year until the lockdown eased (to Level 3 restrictions nationwide) for that Christmas. “Post-adaptation” is thus from the beginning of 2021 when COVID-19 vaccine was available to more people in the country, to mid-2021 when this study was conducted.

#### Pre-Adaptation

In the pre-adaptation phase when most organizations were in preparation for the pandemic, participants highlighted that stress, panic and frustration were evident. These negative feelings stemmed from the unprecedented nature of the pandemic, coupled with unclear information from management. The lack of preparedness for the pandemic is a basic reason, and witnessing panic behaviors of colleagues also exacerbated employees’ stress.

Construction 11: None of us knew anything about COVID and what was going to happen, so people were frustrated that they were still working on site when they didn’t feel like they were safe to be working on site. (FG12)

Local Authority 1: It was madness … there was people bringing in money and putting it in envelopes and locking it in safes and stuff like this totally fishing in the dark at the start. (FG15)

Some OSH representatives worked to provide in-time updates on COVID-19 and its transmission mechanisms, helping to alleviate anxiety and frustration by providing a sense of control. The provision of timely updates, however, requires that organizations be adequately prepared to respond to the public health emergency and faced an onslaught of health and safety queries.

Local Authority 1: we were definitely not ready…all queries seemed to come to health and safety, to see what we were doing and what we could do. (FG15)

Furthermore, even for multinational organizations with access to corporate information sources, extensive emergency scenario planning was not enough to prepare employees psychologically for the pandemic.

Biopharmachem 3: While you can plan for the scenario, it doesn’t fully psychologically prepare people for something that they don’t ever expect to happen. (FG14)

As the poll results presented (Figures 2A,B), 16.9% of the participating organization had redundant employees and 10.2% had to suspend their employees due to the COVID-19 impact. Employees’ pre-adaptation mental health issues were associated with information uncertainty and observed panic behaviors, the solutions to which are discussed in the theme of organizational mental health supports.

#### During Adaptation

With COVID-19 control measures implemented nationwide, most organizations had to adapt their working arrangements to follow new policies. As reported (Figures 2C,D), most participating organizations had no WFH employees or <25% employees who can WFH prior to COVID-19, while the figure was dramatically increased after the emergence of the pandemic. To align with different levels of lockdown restrictions, many employees were sent home to work or were laid off temporarily, especially those who were not essential workers (these workers were able to avail of the government’s pandemic unemployment payment). WFH brought on employee mental health challenges related to isolation/loneliness, worry, fear, internal conflict, and grief.

National Agency 5: … they’re single people, they’re living in a bad s (situation) or something like that. They haven’t even been there, working from an ironing board if (they) have a laptop. (FG5)

Other employees, unable to WFH, were apprehensive about being in close contact with co-workers. Worry was manifested among employees especially in lower-paid positions as they felt financially prohibited from taking time off work. Also, some employees felt stressful WFH when confronting IT (information technology) issues.

Manufacturing 2: we have a lot of people that would [be just] beyond [or] near minimum wage, so they’d be the type that are more inclined to come to work … because they need the money. (FG2)

Infrastructure 2: she’s a cancer survivor and she’s also an asthmatic so you know a lot of issues ... And the IT point of it was really stressful for her. (FG4)

Fear of contracting the virus and giving it to loved ones emerged as another challenge, particularly in instances of advanced age or underlying comorbidities.

Local Authority 3: you’d hear more staff talking about maybe their mother wasn’t well or the father wasn’t well or granny was living in the house, and that they were concerned about that. (FG9)

Concerns for safety were aggravated if an organization was unable to transparently communicate COVID-19 related information, including the number of confirmed cases in the workplace.

Construction 11: If there’s a positive case on a building site [and if] it’s not communicated properly, and the precautions aren’t communicated properly. People really do get nervous, and they got really scared. (FG12)

COVID-19 exemplified the contradictory needs of people between physical health and mental health. People need human contact from the mental perspective, but at the same time people are afraid of the risk of contracting COVID-19 *via* socialization.

Consultant 3: I saw my grandmother for the first time in the 3 months at Christmas and I went to give her a hug, and she was actually terrified that I was coming into her personal space like, you know. I thought the hug it was important, but she wanted to get out of my arm straightway. (FG8)

Finally, workers experienced grief due to loss of co-workers, friends, and family to COVID-19, which was specifically voiced by participants in healthcare.

Healthcare 7: It’s like a war zone, it’s devastating. A lot of carers who work, they’re not there for the money... I mean they’re literally like their grandparents as such. To see staff working [in] homes and they initially are losing 1, 2, 3, 4 not only residents, but sort of their friends, their sort of mentors… it’s just devastating. (FG6)

OSH professionals commented on the absence of ritual or funeral to address the emotions of experiencing loss and grief. Others noted the importance of keeping busy at work to maintain mental wellbeing despite personal loss.

#### Post-Adaptation

Most OSH participants indicated that being on ongoing high-alert for COVID-19 caused constant fear and fatigue for colleagues. As in sectors with high-risk exposure, healthcare workers were particularly under the consistent pressure, and always concerned about the virus transmission.

National Agency 4: …the whole issue of fear was there, you know that you’re that (the person responsible for OSH) and that’s tied into doing the right thing… we’re on high alert and it’s ongoing. (FG7)

Similarly, participants in non-healthcare occupations also described that constant fear could lead to fatigue and inadequate compliance over time with control measures.

Manufacturing 6: … a little bit of fatigue on the facemask wearing as it goes on and goes on, people are getting a little bit more tired of it. (FG6)

More than half of participants indicated that the barrage of negative COVID-19 news in the media exhausted and fatigued their colleagues and themselves. With the emergence of COVID-19 came the emergence of the term “infodemic” [18]. Employees were not able to disconnect with COVID-19 due to the extensive media coverage, especially due to the COVID-19 long-term nature.

Healthcare 1: … they’ve (people) got so many different levels of stress, but not having that time to disconnect and share and offload that piece, absolutely and utterly we’re seeing that [fatigue] (FG5)

Infrastructure 6: … the fatigue part of it, I think they’re mentally drained of the whole thing. (FG4)

Additional factors were highlighted as detrimental to the mental health of employees, such as fatigue caused by children at home during work hours due to school and childcare closures. Details on difficulties finding work-life balance will be discussed in future publications.

### Organizational Mental Health Supports

In addition to sharing perceptions of the mental health impacts of COVID-19 on employees, OSH professionals shared how their organizations worked to alleviate such impacts. Organizations supported mental health of employees by providing timely and reliable information on the management of COVID-19, supports through employee assistance programs (EAPs), by having informal communication channels, allowing for hybrid work and reinforcing control measures in the workplace.

### Providing Timely and Reliable Information on the Management of COVID-19

Since employees’ mental health impacts resulted from uncertainty of the everchanging situation, OSH professionals should provide in-time updates and reliable information to employees in early stages of the pandemic, including transparent communication on positive cases in the workplace.

Infrastructure 1: They were a little panicked at the start were they all going to get it out working on the side of the road or wherever they may have been, and I think the assurances we were given them, and we were explaining how the disease was being transmitted. (FG13)

### Employee Assistance Programs

EAP is a free counselling service supporting employees experiencing work-related or personal problems. Usually, organizations have formal EAPs embedded in their OSH system to support the mental health and wellbeing of employees. OSH participants noted a need for upscaled EAP preparedness and resources to meet the high-volume of demand due to COVID-19.

Local Authority 1: We have a health and wellbeing unit that was initially set up just before COVID got in that was very much in its infancy. For a large organization, we are way behind the curve…we’ve rolled out resilience programs. (FG15)

Participants noted some challenges of their EAPs, including that accessing EAPs required employees to seek out the program themselves, which could limit reach. One participant observed that it was easier for male employees to seek counsel if the OSH professional was a woman.

Construction 4: Being a woman in a male dominated industry, I find men come to women’s talks. I find my office a little bit like a counselling office at sometimes you know. Man to man, you know less likely. (FG11)

### Informal Communication Channels

Informal communication can complement formal EAPs in mitigating the negative impacts of COVID-19 on employee mental health. Unlike EAPs, the use of informal communication can be initiated by OSH professionals. As noted by participants, the key to informal communication is understanding employees’ emotional needs prior to providing them with support. Active listening is an effective approach to gain understanding of an employee’s situation.

Biopharmachem 1: … Try to support them, so you just you kind of had to sit there and let them [talk], just be an ear, more than anything else (FG3)

OSH participants also noted the importance of using informal communication to educate employees on long-term impacts of COVID-19 in order to minimize uncertainty; of initiating proactive communication with employees on potential concerns and stressors and providing a safe environment in which to converse; and of facilitating informal communication between employees themselves. As with EAPs, participants mentioned challenges getting male employees to disclose emotional concerns.

Biopharmachem 2: He broke down and cried. And once I got it out of him, he was fine. It was … building up until he actually broke down and [he said:] ‘I just haven’t seen my daughter! I want to see her [but I cannot] until this company gets it right!’ (FG3)

One positive result of the pandemic was a raised awareness for mental health and wellbeing instead of a workplace culture of “being tough.”

Infrastructure 6: I think it’s very different how people now feel about [it], how people feel they can maybe stand up a little bit more and talk about how they interact and who they trust to interact with. (FG4)

The positive impact of informal communication on employees’ mental health was observed by most participants, the practice of which was simple to perform. Virtual chats, such as online coffee breaks, were also reportedly an effective way.

### Hybrid Work Schedules and Reinforcement of Control Measures

In addition to communication with employees, empirical measures such as hybrid working style and the reinforcement of COVID-19 control measures in the workplace can alleviate employee mental health challenges. A number of organizations realized that the hybrid work has potential benefits for employees’ mental health thanks to increased socialization in the workplace.

Construction 8: A lot of people miss the interaction in instances … a lot of people will come back to the offices when they have the opportunity to do that as well. (FG12)

Returning to work required ongoing reinforcement of COVID-19 control measures to ensure employee safety. Based on the participants’ experience, reinforcing control measures helped combat behavioral fatigue and improve employees’ awareness of COVID-19 safety compliance, as employee adherence reportedly waned over time.

Manufacturing 1: We have a lot of staff… didn’t want to come into work … we had to do a lot of convincing the lads that the site is safe, [that] we have these controls in place and you’re good to come back now. (FG2)

For employees that remained fearful of contracting COVID-19, control measures can alleviate fears by keeping the workplace a safe environment, especially in high-risk.

## Discussion

This study highlighted mental health impacts and associated effects on employees’ adaptation to COVID-19 measures, and strategies used by OSH professionals to mitigate mental health concerns in their workplaces to protect their colleagues. Employees experienced stress, panic and frustration linked to uncertainty and loss of control at the onset of the pandemic, a phenomenon previously identified in the literature [19]. To support employees at this stage and to prevent trust in misleading information sources like social media [20], organizations needed to quickly obtain and communicate reliable information. OSH professionals required adequate support from their organization in order to provide timely and consistent communications to the workforce.

During the transition phase of the pandemic isolation/loneliness and worry were consequences of employee adaptation to increased and ongoing isolation [21] arising from social distancing procedures in the workplace, WFH as well as being laid off during the pandemic at a time of severe social restrictions and lockdowns. Contradictory requirements for maintaining physical and mental health were challenging. Social isolation can be detrimental to mental health but a vital component of COVID-19 safety [22]. Our finding that underlying comorbidities elevated anxiety associated with the virus was expected. An individual’s perception of having poor physical health is associated with higher stress and psychological morbidity [23], as is a history of chronic illness [24]. Some employees required to WFH faced stress stemming from lack of IT skills and/or social supports that would normally be present in their workplaces [25]. Although previous studies emphasized an increased risk for mental health disorders in females during a crisis [26–28], our study shows that male employees were less likely to seek organizational mental health supports or comfort from colleagues, especially in occupations where masculine culture prevailed [29].

Thus, there is a possibility that the manifested increased risk among females is because they are more likely to report mental health impacts and seek for assistance. Gender’s influence on mental health consultations should be considered when planning for public health emergencies, and further research conducted in male dominated industries. Planning for mental health support during a deadly crisis like COVID-19 is of particular importance since restrictions on ritual or funeral may lead to grief and greater risk of prolonged grief disorder [30]. The paid sick leave due to COVID-19 should be advocated to reduce the financial concerns especially for lower-paid employees.

When employees realized the long-term nature of COVID-19 post-adaptation, “pandemic fatigue” set in, referring to the notion of behavioral fatigue associated with adherence to COVID-19 restrictions [31]. Employees’ emotions were dramatically influenced by ongoing negative news from the media, such as daily confirmed cases and deaths reported. To alleviate this, mechanisms for effective communication on evidence-based science to the public would be valuable [18]. Organizations can play a key role in scientific communication during public health emergencies by educating staff, which require the OSH personnel in the organization to be trained for improving such skills.

The findings of this study also reflected that healthcare professionals experienced severe mental health challenges comparing to employees in other occupations. Many studies have focused on COVID-19 mental health issues arising in the healthcare sector due to long hours spent working in high-pressure environments [32]. Suggestions for improving mental health in the healthcare sector include discussions with supervisors who feel confident speaking about mental health [33]; active monitoring for anyone exposed to potentially traumatic events [34]; anonymous online self-check tools; and group discussions to help staff develop a meaningful narrative that reduces risks of harm [35]. Our findings highlight the potential value in expanding these supports to other occupational sectors.

Timely and reliable information from the management regarding COVID-19 become central to the current public health crisis as employees may mistrust information or be confused on conflicting information as a consequence of infodemic [18]. Furthermore, organizations’ mental health programs should incorporate formal and informal communication. Effective formal communication may include confidential consultancy and professional EAP services. Though most organizations provide EAP within their OSH program, we found that not all EAPs were equipped to meet the large-scale demands of a public health emergency. However, the efficacy of EAP may be limited in male dominated industries where stigma prevents employees from seeking out services [36]. Informal communication by comparison can be initiated by OSH professionals and is an effective mechanism for mitigating mental health impacts. Informal chats can be arranged after a formal online meeting by giving employees space to talk about non-work-related topics.

Finally, hybrid working arrangements can alleviate employee isolation and loneliness by increasing opportunities for social interaction and informal communication. As employees return to work, reinforced occupational control measures are vital for protection and reassurance. Meanwhile, the employer should be open to changes depending on the situation, such as acceptance of the possibility that employees need to remain WFH if COVID-19 risk becomes high. Nevertheless, occupational COVID-19 controls can have a negative impact on employees’ mental health if they see the stringency of control measures as an indicator of high risk at work [37]. It is important that management provides transparent communications to ensure that employees maintain an accurate perception of COVID-19 contagion risk at work, such as email updates with links to reliable sources.

### Conclusion

This is the first qualitative study conducted with OSH/HR professionals in Ireland regarding COVID-19 adaptation, which indeed provide valuable insights to the research literature, as well as empirical experience in supporting employees with mental health impacts arising from pandemic workplace adaptation measures. As a limitation, participants’ opinions may not be sufficiently representative because of the qualitative study nature (e.g., the baseline assumption about the stress level may vary in different occupational settings). Future research focusing on specific occupation(s) especially high-risk sectors (e.g., healthcare) is needed to provide in-depth information for the development of intervention program customized to specific working setting(s). However, qualitative research design in this study allows for richer data to be gathered than a quantitative survey at a time when significant occupational and societal flux was in progress due to the changing nature of the pandemic’s early stages. To complement this, there is a need for the design of a quantitative survey instrument to investigate the COVID-19 adaptation challenges from more employees. Such a survey is under development in our project, which will enable the OSH professionals to have a better understanding of their employees’ needs during the current and future public health emergencies, and thus customize their support to specific mental health issues in their organization.
